# Prevalence and prognostic value of various types of right ventricular dysfunction in mechanically ventilated septic patients

**DOI:** 10.1186/s13613-021-00902-9

**Published:** 2021-07-13

**Authors:** Hongmin Zhang, Wei Huang, Qing Zhang, Xiukai Chen, Xiaoting Wang, Dawei Liu, Critical Care Ultrasound Study Group

**Affiliations:** 1 Department of Critical Care Medicine, Peking Union Medical College Hospital, Chinese Academy of Medical Sciences and Peking Union Medical College, 1# Shuai Fu Yuan, Dong Cheng District, Beijing, 100730 China; 2 Pittsburgh Heart, Lung, Blood and Vascular Institute, University of Pittsburgh, School of Medicine, Pittsburgh, PA USA

**Keywords:** Right ventricular failure, Right ventricular systolic dysfunction, Sepsis, Prognosis

## Abstract

**Introduction:**

Right ventricle (RV) dilation in combination with elevated central venous pressure (CVP), which is a state of RV congestion, is seen as a sign of RV failure (RVF). On the other hand, RV systolic function is usually assessed by tricuspid annular plane systolic excursion (TAPSE) and fractional area change (FAC). This study aimed to investigate the prevalence and prognostic value of RVF and RV systolic dysfunction (RVSD) in septic patients.

**Methods:**

Mechanically ventilated sepsis and septic shock patients were included. We collected haemodynamic and echocardiographic parameters as well as prognostic information including mechanical ventilation duration, length of ICU stay and 30-day mortality. RVF was defined as a right and left ventricular end-diastolic area ratio ≥ 0.6 in combination with CVP ≥ 8 mmHg. RVSD was defined as TAPSE < 16 mm or FAC < 35%.

**Results:**

A total of 215 patients were enrolled in this study, and the patients were divided into 4 groups: patients with normal RV function (normal, *n* = 101), patients with RVF but without RVSD (RVF only, *n* = 38), patients with RVSD but without RVF (RVSD only, *n* = 44), and patients with combined RVF–RVSD (RVF/RVSD, *n* = 32). The RVF/RVSD group and RVSD only group had a lower cardiac index than the RVF only group and normal groups (*p* < 0.05). At 30 days after ICU admission, 50.0% of patients had died in the RVF/RVSD group, which was much higher than the mortality in the RVF only group (13.2%) and normal group (13.9%) (*p* < 0.05). In a Cox regression analysis, the presence of RVF/RVSD was independently associated with 30-day mortality (HR 3.004, 95% CI:1.370–6.587, *p* = 0.006). In contrast, neither the presence of RVF only nor the presence of RVSD only was associated with 30-day mortality (HR 0.951, 95% CI:0.305–2.960, *p* = 0.931; HR 1.912, 95% CI:0.853–4.287, *p* = 0.116, respectively).

**Conclusion:**

The presence of combined RVF–RVSD was associated with 30-day mortality in mechanically ventilated septic patients. Additional studies are needed to confirm and expand this finding.

**Supplementary Information:**

The online version contains supplementary material available at 10.1186/s13613-021-00902-9.

## Introduction

Sepsis is a major public concern and the leading cause of mortality in critically ill patients [1, 2]. Myocardial dysfunction is common in sepsis patients and can involve the left ventricle (LV) as well as the right ventricle (RV) [3, 4]. Unlike the LV, the geometry of the RV is complex, and RV longitudinal strain and 3D echo are not readily available in the intensive care unit (ICU) [5]. Thus, tricuspid annular plane systolic excursion (TAPSE), and fractional area change (FAC) remain the most commonly used quantitative parameters of RV systolic function [6, 7].

RV dilation is usually represented by the ratio of RV and LV end-diastolic areas [8, 9]. Vieillard-Baron and his colleagues contended that RV dilation in combination with elevated central venous pressure (CVP) was a state of RV congestion and could unmask the occurrence of RV failure (RVF). They reported that RVF was more sensitive than TAPSE in the assessment of volume responsiveness in septic shock patients [10]. Prior studies have proven that RV dysfunction is associated with long-term prognosis in septic patients [11–13]. However, whether RVF, diagnosed by RV dilation and elevated CVP, was also of prognostic value has not been reported. Therefore, we performed this study to investigate the prevalence of RVF and RV systolic dysfunction (RVSD) and their association with cardiac output, ICU stay and 30-day mortality in mechanically ventilated septic patients.

## Patients and methods

### Study population

This study was an observational study conducted at a tertiary hospital’s intensive care unit (ICU). We retrospectively studied a cohort of adult septic patients who were on mechanical ventilation from 1 May 2018 to 1 August 2020.

We adopted the same definition of sepsis and septic shock as described in Sepsis-3 [14]. The exclusion criteria included the following: lack of CVP monitoring; lack of MV support via tracheal intubation; intra-abdominal pressure above 12 mmHg; new onset of acute coronary syndrome within 1 week; severe valvular disease or history of valvular surgery; history of chronic pulmonary hypertension; insufficient echocardiographic image; and withholding of life support.

### Echocardiography

Echocardiograms were recorded within the first 24 h of ICU admission. Two physicians (H Zhang and Q Zhang) with 10 years of echo experience obtained the images and they were blinded to the clinical states of the patients upon echo examination. The echo results were reported based on the PRICES statement [15]. At least three cardiac cycles were analysed and averaged. M-mode and Doppler echocardiographic measurements were taken according to standard protocols. The measurement of TAPSE, left ventricular ejection fraction (LVEF), averaged tissue Doppler velocity of lateral and medial mitral annuli at early diastole (e’), and tricuspid regurgitation (TR) were performed as previously described [12]. The ratio of RV end-diastolic area and LV end-diastolic area (R/LVEDA) was obtained at the end of ventricular diastole. FAC was defined as (end-diastolic area – end-systolic area)/end-diastolic area × 100 [6]. The E velocity was measured using pulsed wave Doppler with the sample volume placed between the tips of the mitral valve. The diameter of the left ventricular outflow tract (LVOT) was obtained at the parasternal long-axis view. The velocity–time integral (VTI) was obtained by positioning the sample volume at the LVOT approximately 0.5 cm below the aortic valve via pulsed Doppler imaging [16]. Cardiac output (CO) was calculated using the following formula: CO = π × (LVOT diameter/2)^2^ × VTI × heart rate. The CO was then indexed to body surface area.

### Other parameters collected

We collected the patients’ demographic information, Acute Physiology and Chronic Health Evaluation (APACHE) II score, and Sequential Organ Failure Assessment (SOFA) score at ICU admission. Each patient’s heart rate (HR), mean arterial pressure (MAP), CVP, positive end-expiratory pressure (PEEP), and plateau pressure (Pplat) were also collected at the time of the echo examination.

#### Definition

RVF was defined as R/LVEDA ≥ 0.6 in combination with CVP ≥ 8 mmHg according to a recent study by Vieillard-Baron et al. [10]. RVSD was defined as TAPSE < 16 mm or FAC < 35% [6, 17].

### Outcomes

The primary outcome was 30-day survival, and the secondary outcomes included length of ICU stay, mechanical ventilation (MV) duration and cardiac index.

### Statistical analysis

We performed statistical analysis using SPSS 13.0 (SPSS, Inc., Chicago, Illinois, USA). Continuous variables are expressed as the mean ± SD or as the median and the interquartile range. Categorical variables are presented as frequencies and percentages. The distributions of the continuous values were assessed for normality by the Kolmogorov–Smirnov test. Differences among groups were assessed by one-way ANOVA, the Kruskal–Wallis test, the Chi-squared test, or Fisher’s exact test, as appropriate. If necessary, a Dunn–Bonferroni test was performed for post hoc comparisons. Receiver operating characteristic (ROC) curves were generated and the areas under each respective curve were calculated. Prognostic factors for 30-day mortality were determined using the Cox regression model. The following variables were considered for the survival analysis: age, SOFA score, APACHE II score, PEEP, Pplat, LVEF, E/e’, RVF and RVSD. The variables that had *p* < 0.1 in the univariable model were included in the multivariable model and the hazard ratio was calculated, together with its 95% confidence interval. Cumulative survival curves of the 30-day follow-up were estimated with the Kaplan–Meier method. Sensitivity analyses were performed using different cut-off values for RVF, as well as incorporating LV systolic dysfunction, when investigating the association between RV function and 30-day mortality. Intraobserver and interobserver variabilities in LVEF, TR velocity and FAC were assessed in 20 randomly selected patients and were tested using intraclass correlation coefficients (ICCs). An ICC > 0.8 was considered excellent agreement. Two-tailed *p* < 0.05 was considered significant.

## Results

### General characteristics

**Fig. 1:**
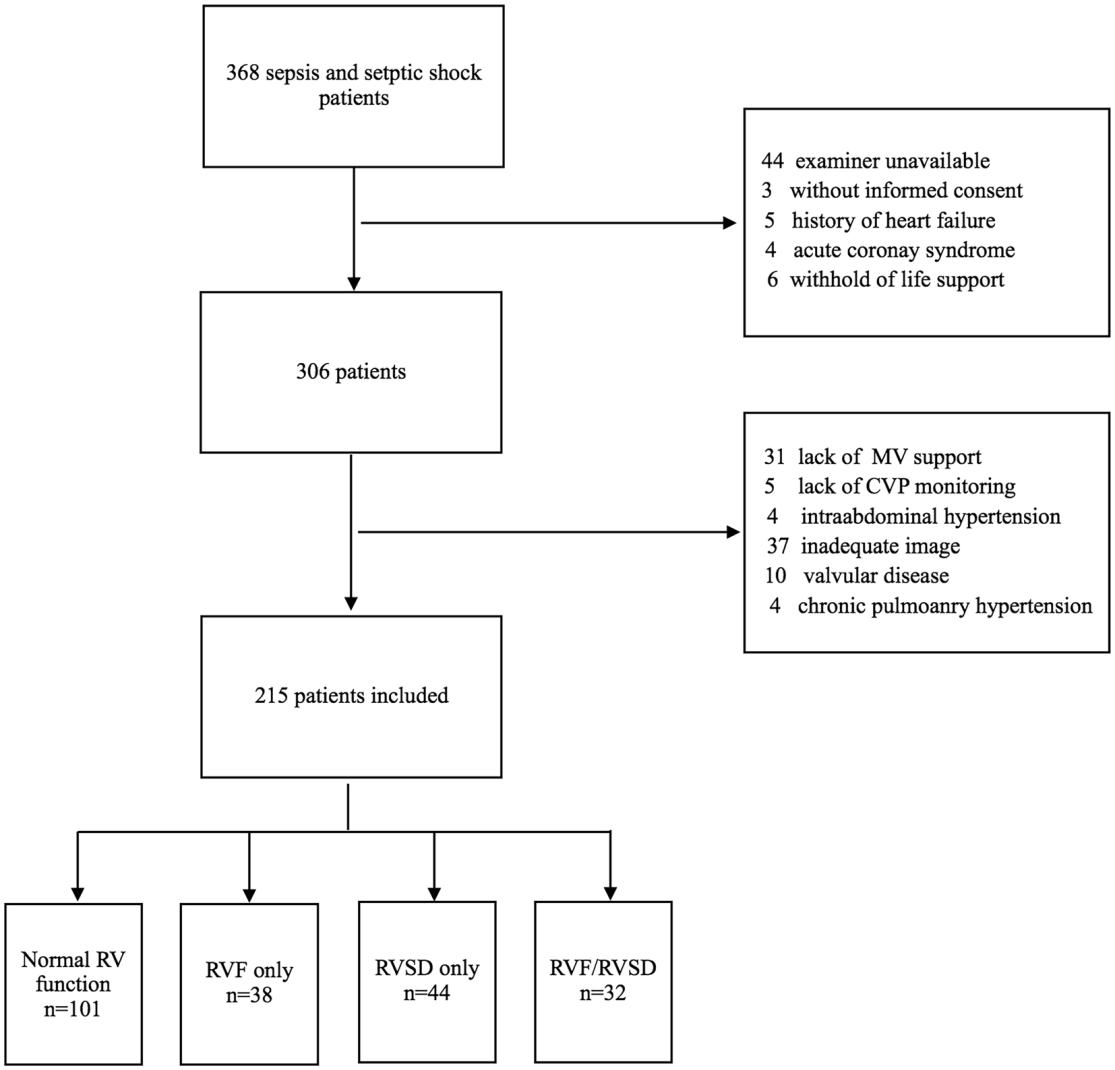
Flowchart of the study. *MV* mechanical ventilation, *CVP* central venous pressure, *RV* right ventricle, *RVF only* patients with RV failure but without RV systolic dysfunction, *RVSD only* patients with RVSD but without RV failure; *RVF/RVSD* patients with combined RVF–RVSD

In all, 368 patients were screened for enrolment, and 215 patients were included in this study. The patients were divided into 4 cohorts based on the presence of RVF and RVSD: patients with normal RV function (normal, *n* = 101), patients with RVF but without RVSD (RVF only, *n* = 38), patients with RVSD but without RVF (RVSD only, *n* = 44), and patients with combined RVF–RVSD (RVF/RVSD, n = 32) (Fig. 1). The general characteristics are listed in Additional file 1: Tables S1 and S2.

The four groups had similar age and sex proportions. The RVF/RVSD group had higher APACHE II and SOFA scores than the RVF and normal groups (*p* < 0.05). The RVF/RVSD group had the highest PEEP level among all groups (*p* = 0.001) (Table 1).

**Table 1:** Demographics, illness severity, and haemodynamic and echocardiographic findings

Categories	All patients (*n* = 215)	Normal (*n* = 101)	RVF only (*n* = 38)	RVSD only (*n* = 44)	RVF/RVSD (*n* = 32)	*p* value
Age (year)	65 (50, 73)	64 (50, 73)	65 (47, 74)	64 (57, 75)	65 (51, 74)	0.774
Sex (male, %)	134 (62.3%)	61 (60.4%)	24 63.2%)	28 (63.6%)	21 (65.6%)	0.104
APACHE II	20 (15, 26)	19 (13, 26)	19 (14, 24)	22 (17, 27)	24 (18, 30)	0.043^b, c^
SOFA	12 (9, 14)	11 (8, 13)	12 (8, 13)	13 (10, 15)	14 (11, 17)	0.007^b, c, e^
HR (bpm)	94 ± 20	93 ± 16	88 ± 21	98 ± 22	97 ± 22	0.109
MAP (mmHg)	76 (66, 82)	75 (66, 85)	77 (71, 84)	76 (66, 83)	72 (68, 76)	0.268
CVP (mmHg)	9 (7, 11)	8 (6, 10)	9 (8, 11)	8 (7, 10)	10 (8, 12)	< 0.001^a, c, f^
PEEP (cmH_2_O)	5 (5, 8)	5 (5, 6)	5 (5, 7)	6 (5, 8)	6 (5, 10)	0.001^b, c, e^
Pplat (cmH_2_O)	18 (16, 22)	18 (16, 21)	19 (15, 22)	18 (16, 20)	20 (18, 23)	0.097
*Fluid before echo (ml)	3764 (3206, 4589)	3722 (2677, 4704)	3701 (3193, 4471)	3773 (3326, 4598)	3884 (2945, 4804)	0.992
R/LVEDA	0.55 (0.45, 0.65)	0.49 (0.43, 0.55)	0.67 (0.63, 0.71)	0.45 (0.39, 0.53)	0.68 (0.63, 0.72)	< 0.001^a, c, d, f^
TAPSE (mm)	19.0 ± 5.1	21.8 ± 3.6	21.5 ± 4.0	14.0 ± 2.9	14.0 ± 3.3	< 0.001^b, c, d, e^
FAC (%)	46 (38, 52)	49 (44, 55)	48 (41, 55)	34 (29, 47)	32 (29, 44)	< 0.001^b, c, d, e^
TR (m/s)	2.4 ± 0.5	2.4 ± 0.3	2.4 ± 0.4	2.4 ± 0.5	2.6 ± 0.5	0.112
LVEF (%)	60 (50, 69)	62 (56, 70)	63 (54, 69)	55 (44, 62)	52 (47, 63)	< 0.001^c, d, e^
E/e’	8.5 (6.6, 10.7)	8.0 (6.6, 10.0)	7.8 (6.5, 10.8)	8.9 (6.7, 12.2)	9.8 (5.7, 13.5)	0.060
CI (L/min/m^2^)	3.4 (2.8, 4.0)	3.6 (3.0, 4.2)	3.4 (2.9, 4.3)	3.1 (2.5, 3.8)	3.1 (2.7, 3.7)	< 0.001^c, d, e^
MV duration (hr)	100 (36, 235)	91 (30, 232)	93 (30, 172)	105 (67, 211)	138 (62,282)	0.379
ICU stay (day)	6 (3, 12)	6 (3, 11)	4 (3, 10)	7 (4, 12)	7 (3, 14)	0.086
30-day mortality (*n*, %)	50 (23.2%)	14 (13.9%)	5 (13.2%)	15 (34.1%)	16 (50%)	< 0.001^b, c^

*Fluid administered within 24 h before echo examination

^a^RVF/RVSD vs. RVSD, *p* < 0.05; ^b^RVF/RVSD vs. RVF, *p* < 0.05; ^c^RVF/RVSD vs. Normal, *p* < 0.05; ^d^RVSD vs. RVF, *p* < 0.05; ^e^RVSD vs. Normal, *p* < 0.05; ^f^RVF vs. Normal, *p* < 0.05

*APACHE* Acute Physiology and Chronic Health Evaluation, *SOFA* Sequential Organ Failure Assessment, *HR* heart rate, *MAP*: mean arterial pressure, *CVP* central venous pressure, *PEEP* positive end-expiratory pressure, *Pplat* plateau pressure, *R/LVEDA* ratio of right and left end-diastolic area, *TAPSE* tricuspid annular plane systolic excursion, *FAC* fractional area change, *TR* tricuspid regurgitation, *LVEF* left ventricular ejection fraction, *CI* cardiac index, *MV* mechanical ventilation, *ICU* intensive care unit

### Comparison of haemodynamic and echocardiographic parameters

The intraobserver variability analysis revealed that the ICCs for LVEF, TR velocity and FAC were 0.908 (95% CI: 0.782–0.962), 0.916 (95% CI: 0.801–0.966), and 0.851 (95% CI: 0.661–0.938), respectively. The interobserver variabilities for LVEF, TR velocity and FAC were 0.875 (95% CI: 0.712–0.949), 0.904 (95% CI: 0.758–0.962), and 0.827 (95% CI: 0.614–0.928), respectively.

**Fig. 2:**
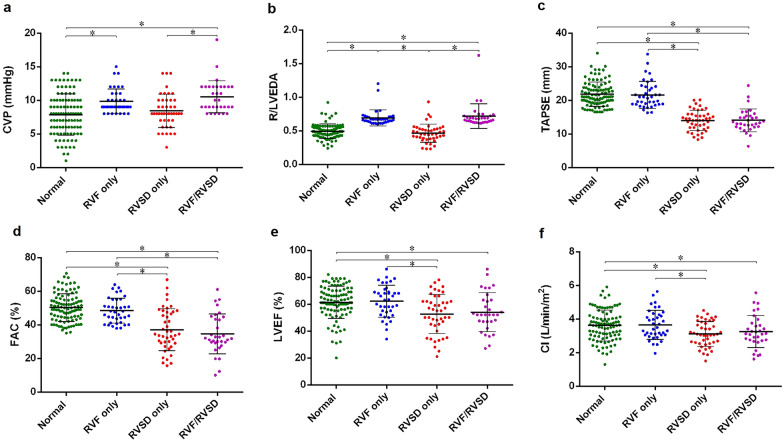
Haemodynamic and echocardiographic parameters in four groups. **a** RVF/RVSD group had higher CVP than RVSD only group and normal group (*p* < 0.005). RVF only group had higher CVP than normal group (*p* < 0.005). **b** RVF/RVSD group and RVF only group had higher R/LVEDA ratio than RVSD only group and normal group (*p* < 0.05). **c** RVF/RVSD group and RVSD only group had lower TAPSE than RVF only group and normal group (*p* < 0.05). **d** RVF/RVSD group and RVSD only group had lower FAC than RVF only group and normal group (*p* < 0.05). **e** RVF/RVSD group and RVSD only group had lower LVEF than normal group (*p* < 0.05). **f** RVF/RVSD group and RVSD only group had lower CI than RVF only group and normal group (*p* < 0.05)

The four groups had similar HR and MAP. Both the RVF/RVSD group and RVSD only groups had a lower LVEF than the normal group (*p* < 0.05) (Table 1, Fig. 2e).

### Primary outcome

At 30 days after ICU admission, 50.0% of patients had died in the RVF/RVSD group, which was much higher than the mortality in the RVF only group (13.2%) and normal groups (13.9%) (*p* < 0.05). The mortality in the RVF/RVSD group was also higher than that in the RVSD group (34.1%), but was not statistically significant.

**Fig. 3:**
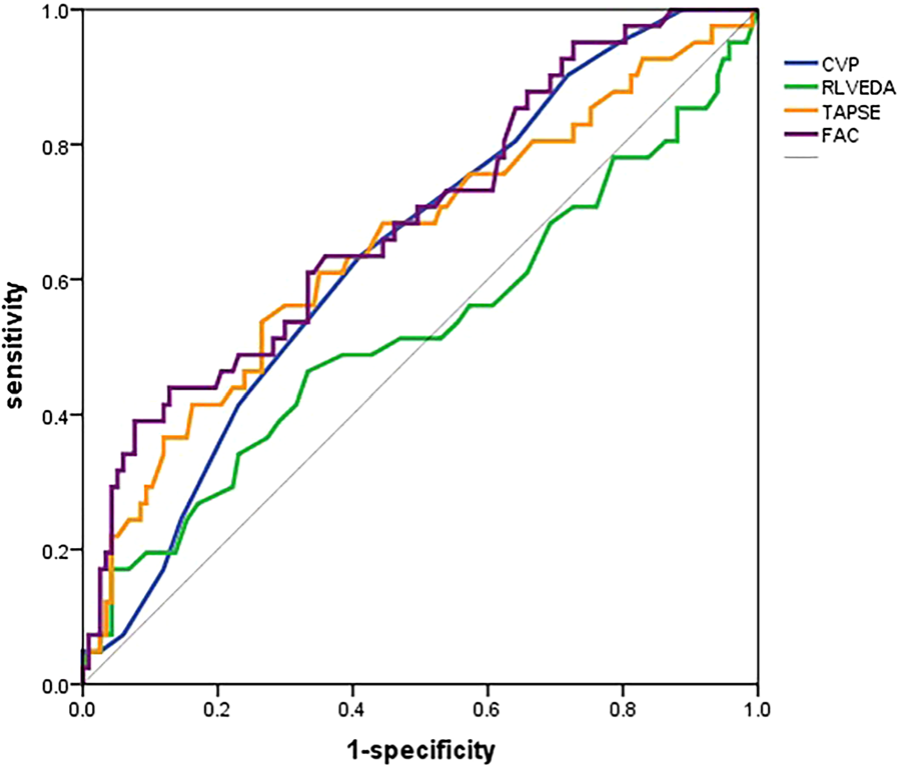
ROC curve analysis of CVP, R/LVEDA, TAPSE, and FAC for 30 day mortality. *CVP* central venous pressure, *R/LVEDA* ratio of right and left ventricular end-diastolic area, *TAPSE* tricuspid annular plane systolic excursion, *FAC* fractional area change. The ROC analysis showed that the area under the curve for CVP, R/LVEDA, TAPSE and FAC were 0.644, *p* = 0.006; 0.525, p = 0.634; 0.652, *p* = 0.004 and 0.690, *p* < 0.001, respectively

The ROC analysis showed that the areas under the curve for CVP, R/LVEDA, TAPSE and FAC were 0.644 (*p* = 0.006); 0.525 (*p* = 0.634); 0.652 (*p* = 0.004) and 0.690 (*p* < 0.001), respectively (Fig. 3, Table 2).

**Table 2:** ROC analysis of variables for the prediction of 30-day mortality

Categories	AUC	95% CI	*p*	Optimum cut-off	Sen	Spe	PPV	NPV
CVP (mmHg)	0.644	0.551–0.737	0.006	8.5	63.4	59.0	32.0	84.1
R/LVEDA	0.525	0.413–0.637	0.634	–	–	–	–	–
TAPSE (mm)	0.652	0.550–0.754	0.004	18.1	61.0	65.0	34.6	84.6
FAC (%)	0.690	0.595–0.786	< 0.001	44	61.0	66.7	35.8	84.9

*CVP* central venous pressure, *R/LVEDA* ratio of right and left end-diastolic area, *TAPSE* tricuspid annular plane systolic excursion, *FAC* fractional area change

**Fig. 4:**
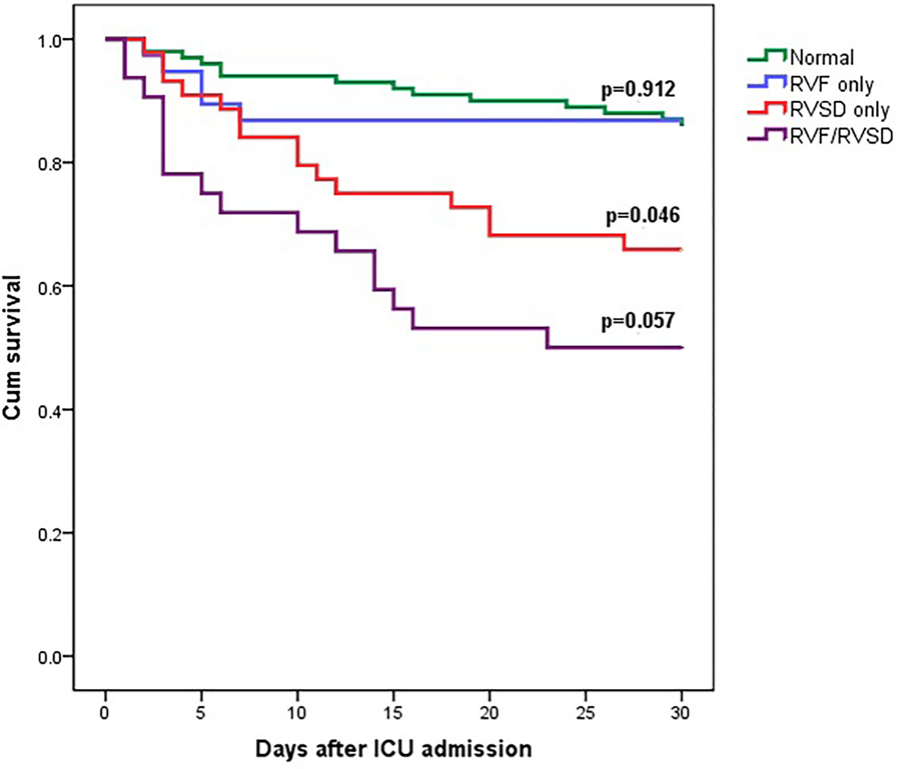
The Kaplan–Meier curves for estimated survival analysis. The RVF/RVSD group had the highest mortality (RVF/RVSD vs. RVSD only, log-rank:3.662, *p* = 0.057; RVF/RVSD vs. RVF only, log-rank:12.613, *p* < 0.001; RVF/RVSD vs. normal, log-rank:25.208, *p* < 0.001); The RVSD only group had higher mortality than the RVF only and normal groups (RVSD only vs. RVF only, log-rank:3.995, *p* = 0.046; RVSD only vs. normal, log-rank: 7.376, *p* = 0.007); No difference was found between the RVF only group and the normal group (RVF only vs. Normal, log-rank: 0.012, *p* = 0.912). *RVF only* patients with RV failure but without RV systolic dysfunction, *RVSD only* patients with RVSD but without RV failure, *RVF/RVSD* patients with combined RVF–RVSD

We generated Kaplan–Meier curves for estimated survival at 30 days after ICU admission. The RVF/RVSD group had higher mortality than the RVF only and normal groups (RVF/RVSD vs. RVF only, log-rank:12.613, *p* < 0.001; RVF/RVSD vs. normal, log-rank:25.208, *p* < 0.001). The RVF/RVSD group also had higher mortality than the RVSD only group, but was not statistically significant (RVF/RVSD vs. RVSD only, log-rank:3.662, *p* = 0.057). The RVSD only group had higher mortality than the RVF only group and normal groups (RVSD only vs. RVF only, log-rank:3.995, *p* = 0.046; RVSD only vs. normal, log-rank: 7.376, *p* = 0.007). No difference was found between the RVF only group and normal groups (RVF only vs. normal, log-rank: 0.012, *p* = 0.912) (Fig. 4).

In a Cox regression survival analysis, after adjusting for APACHEII, SOFA, PEEP, Pplat, and E/e’, the presence of RVF/RVSD was independently associated with 30-day mortality (HR 3.004, 95% CI: 1.370–6.587, *p* = 0.006). In contrast, neither the presence of RVF only nor the presence of RVSD only was associated with 30-day mortality (HR 0.951, 95% CI: 0.305–2.960, *p* = 0.931; HR 1.912, 95% CI:0.853–4.287, *p* = 0.116) (Table 3).

**Table 3:** Factors associated with 30-day mortality with sensitivity analysis

	Hazard ratio	95% CI	*p* value
Univariable analysis			
Age	1.006	0.989–1.024	0.478
APACHEII	1.073	1.038–1.110	< 0.001
SOFA	1.230	1.134–1.334	< 0.001
PEEP	1.185	1.078–1.302	< 0.001
Pplat	1.108	1.061–1.157	< 0.001
Fluid before echo	1.002	0.991–1.016	0.127
LVEF	1.001	0.980–1.023	0.904
E/e’	1.063	1.013–1.115	0.013
CI	0.857	0.644–1.142	0.293
RVF only	0.501	0.199–0.263	0.143
RVSD only	1.815	0.991–3.325	0.054
RVF/RVSD	2.960	1.615–5.426	< 0.001
Multivariable analysis			
SOFA	1.112	1.012–1.223	0.028
Pplat	1.115	1.052–1.182	< 0.001
RVF only	0.951	0.305–2.960	0.931
RVSD only	1.912	0.853–4.287	0.116
RVF/RVSD	3.004	1.370–6.587	0.006

*APACHE* Acute Physiology and Chronic Health Evaluation, *SOFA* Sequential Organ Failure Assessment, *CVP* central venous pressure, *PEEP* positive end-expiratory pressure, *Pplat* plateau pressure, *LVEF* left ventricular ejection fraction, *CI* cardiac index, *RVC only* patients with RV congestion but without RV systolic dysfunction, *RVSD only* patients with RVSD but without RV congestion, *RVC/RVSD* patients with both RVC and RVSD

### Sensitivity analysis

We performed sensitivity analysis by using CVP ≥ 10 mmHg, CVP ≥ 12 mmHg, and R/LEDA ≥ 0.7 separately as cut-off values of RVF, and found that RVF was still not an independent predictor of 30-day mortality in these patients.

### Secondary outcomes

The RVF/RVSD and RVSD only groups had a lower cardiac index than the RVF only group and normal groups (*p* < 0.05). No significant difference was found regarding MV duration or length of ICU stay among the four groups (Table 1, Fig. 2f).

## Discussion

In this study, we investigated the prevalence of RVF and RVSD and their association with short-term mortality in mechanically ventilated septic patients. We found that the presence of combined RVF–RVSD was associated with 30-day mortality. Neither RVF nor RVSD, when occurring alone, was a predictor of 30-day mortality in these patients.

The definition and criteria of RVF reported by Vieillard-Baron are reasonable, which provides physicians with a new perspective for RV function evaluation. An acute elevation in RV preload or afterload is manifested with RV dilation, which can be quickly estimated by R/LVEDA [7, 8]. On the other hand, the primary function of the RV is to keep CVP as low as possible [18]. When the RV fails, CVP will rise inevitably. Therefore, the diagnosis of RVF based on RV dilation and CVP makes sense in the appraisal of RV function.

Although CVP was one of the criteria to define RVF, our results revealed that the CVP values among the four groups, given significant differences, were very close. In comparison with study by Vieillard-Baron, the R/LVEDA in this study was smaller (interquartile 0.63–0.72 vs. 0.7–0.9), which might partly explain this result [10]. Next, the CVP is the intramural pressure rather than the transmural pressure of the RV, while the actual pressure that determines RV preload is the CVP relative to the pressure surrounding the heart [19–22]. We cannot exclude conditions where the transmural pressure was normal, while CVP increased due to elevated pleural pressure. Furthermore, LV systolic function is often compromised in septic patients, and a concomitant RV dysfunction might ensue, probably because the LV contributes 30% of the contraction force to RV systolic function [3, 23, 24]. In this case, R/LVEDA might not be enough to diagnose RV enlargement. If the LV is dilated, RV size may be underestimated, and quantification of RV size should be performed independently to determine if there is RV dilation [18, 25]. Therefore, we suppose that the inherent definition of RVF might result in the overlap of CVP among the four groups, which hopefully would justify the combination of RVF and RVSD in the evaluation of RV.

This study found that RVF alone was not associated with 30-day mortality. Several reasons might help explain this finding. First, the cut-off value of CVP to detect RVF was relatively low. The recommended range of CVP was from 8 to 12 mmHg, or even 12–15 mmHg for patients on mechanical ventilation in the SSC guidelines [26]. The interagency Registry for Mechanically Assisted Circulatory Support defines RV failure as an elevated CVP ˃16 mmHg and end-organ dysfunction [27]. Second, we did not notice a significant decrease in the cardiac index of the RVF only group. The patients seemed to be in a state of systemic congestion without compromise of cardiac output. An acute increase in either preload or afterload is immediately associated with RV dilation [28, 29]. No significant difference in TR was found between patients with RVF only and normal patients. Thus, we supposed that the preload (rather than afterload) was responsible for RV dilation in the RVF only group.

RVF and RVSD can occur separately and collectively. A recent study pointed out that RVSD was associated with 28-day mortality in septic patients [17]. However, they did not mention the presence of RVF. It was not clear whether RVSD was still a predictor of mortality if patients with RVF were excluded from their study. This study found that patients with combined RVF–RVSD had the highest mortality. We hypothesized that various types of RV involvement could provide clues about the severity of RV dysfunction (i.e. RVF indicates a lower chance of volume responsiveness, RVSD indicates a higher chance of a decreased cardiac index, and the combination of RVF and RVSD signifies a worse prognosis). Additional research is still warranted in terms of this RV function classification, but we believe it would be clinically relevant.

## Limitations

This study has several limitations. First, given the nature of the retrospective analysis, we did not assess the volume responsiveness of these patients. In future studies, the assessment of volume responsiveness of RVSD patients should be considered and might add value to the classification of RV function. Second, the follow-up was not long enough. We are not certain about the association between RV dysfunction and long-term prognosis. Furthermore, we chose the initial examination to predict outcome when treatment and response had not occurred. We think a series of echo examinations would yield more robust evidence. Third, RV dilation in combination with septal paradoxical motion can easily assess RV function in a qualitative way [8]. We did not collect information about the septum, which might provide clues about the volume or pressure overload of the RV. Fourth, we only included patients on mechanical ventilation, and the conclusion cannot be applied to spontaneously breathing patients. Last, we had to admit that the terms “RVF only” and “RVSD only” were not perfect. However, we avoided choosing the term “isolated RVF” or “isolated RVSD”, which might cause confusion.

## Conclusion

The presence of combined RVF–RVSD was associated with 30-day mortality in mechanically ventilated septic patients. Additional studies are needed to confirm and expand this finding.

## Supplementary Information

**Additional file 1:** **Table S1.** General characteristics of all patients. **Table S2.** Factors associated with 30-day mortality.

## Data Availability

All datasets used and analysed during the current study are available from the corresponding author on reasonable request.

## Abbreviations

RV: Right ventricle
LV: Left ventricle
RVF: Right ventricular failure
RVSD: Right ventricular systolic dysfunction
TAPSE: Tricuspid annular plane systolic excursion
FAC: Fractional area change
CVP: Central venous pressure
ICU: Intensive care unit
MV: Mechanical ventilation
LVEF: Left ventricular ejection fraction
TR: Tricuspid regurgitation
R/LVEDA: The ratio of RV end-diastolic area and LV end-diastolic area
LVOT: Left ventricular outflow tract
VTI: Velocity–time integral
APACHE: Acute Physiology and Chronic Health Evaluation
SOFA: Sequential Organ Failure Assessment
HR: Heart rate
MAP: Mean arterial pressure
PEEP: Positive end-expiratory pressure
Pplat: Plateau pressure
CI: Cardiac index
RVF/RVSD: Presence of combined RVF–RVSD
