# Supplementary material for: Prevalence and prognostic value of various types of right ventricular dysfunction in mechanically ventilated septic patients
Source: Ann Intensive Care. 2021 Jul 13;11:108. doi: 10.1186/s13613-021-00902-9 (PMC8276910; doi:10.1186/s13613-021-00902-9)
Supplement: Supplementary file 1 — Additional file 1: Table S1. General characteristics of all patients. Table S2. Factors associated with 30-day mortality. [file 13613_2021_902_MOESM1_ESM.docx]

Supplemental table 1. General characteristics of all patients

| Categories | Findings (n=215) |
| --- | --- |
| Age (yr) | 65 (50, 74) |
| Sex (male, %) | 128 (59.5%) |
| APACHE Π | 20 (15, 26) |
| SOFA | 12 (9, 14) |
| Diagnosis (n, %) |  |
| Abdominal infection | 120 (55.8%) |
| Pneumonia | 44 (20.5%) |
| UTI | 8 (3.7%) |
| Skin and soft tissue | 19 (8.8%) |
| Bloodstream infection | 7 (3.3%) |
| Others* | 17 (7.9%) |
| Comorbidities |  |
| HTN | 109 (50.7%) |
| DM | 83 (38.6%) |
| CAD | 63 (29.3%) |
| CKD | 32 (14.9%) |
| COPD | 20 (9.3%) |
| Septic shock (n, %) | 144 (70.0%) |
| ARDS (n, %) | 39 (18.1%) |
| NE infusion (n, %) | 170 (79.0%) |
| NE dose (μg/kg/min) | 0.37 (0.15, 0.71) |
| Lactate (mmol/L) | 2.4 (1.6, 3.8) |

*Others including intracranial, mediastinum infections and infections without confirmed source.

APACHE: acute physiology and chronic health evaluation; SOFA: sequential organ failure assessment; CRBSI: catheter related bloodstream infection; HTN: hypertension; DM: diabetes mellitus; CAD: coronary arterial disease; CKD: chronic kidney dysfunction; COPD: chronic obstructive pulmonary disease; NE: norepinephrine; ARDS: acute respiratory distress syndrome.

Supplemental table 2. Factors associated with 30-day mortality

|  | Hazard Ratio | 95%CI | *p* Value |
| --- | --- | --- | --- |
| Multivariable analysis |  |  |  |
| RVC (CVP≥10) | 1.455 | 0.696-3.042 | 0.319 |
| RVC (CVP≥12) | 0.811 | 0.312-2.108 | 0.668 |
| RVC (R/VEDA≥0.7) | 1.410 | 0.543-4.467 | 0.557 |
| Interaction |  |  |  |
| Pplat*RVSD | 1.102 | 0.966-1.257 | 0.147 |
| Pplat*RVC | 1.073 | 0.959-1.201 | 0.217 |

R/LVEDA: ratio of right and left end-diastolic area; TAPSE: tricuspid annular plane systolic excursion; FAC: fractional area change; PASP: pulmonary arterial systolic pressure; SVI: stroke volume index; CI: cardiac index;
